# Death Anxiety and Resilience in Older Adults: The Moderating Role of Attachment Patterns

**DOI:** 10.3390/bs14111031

**Published:** 2024-11-04

**Authors:** Yoav S. Bergman

**Affiliations:** Faculty of Social Work, Ashkelon Academic College, 12 Ben Tzvi St., Ashkelon 78211, Israel; yoav.s.bergman@gmail.com; Tel.: +972-54-2124400

**Keywords:** attachment theory, death anxiety, older adults, resilience, terror management theory

## Abstract

The unique human awareness of the fact that life is finite, and that death is unavoidable has been shown to elicit negative psychological consequences across the life cycle. However, research has demonstrated that the ability to seek, maintain, and gain comfort from close relationships mitigates the adverse psychological effects of death awareness/anxiety. Moreover, relatively little is known about how death anxiety and social relationships in old age are associated with resilience, an important personal protective factor for dealing with general and age-related difficulties. Accordingly, the current work examined the links between death anxiety and resilience in older adults and explored the potential moderating role of attachment patterns for this link. Data were collected from 369 older Israeli adults (*M*age = 73.15, *SD* = 6.31, range = 60–94), who completed scales examining death anxiety, attachment patterns, and resilience, as well as sociodemographic scales. The results demonstrated that death anxiety, attachment anxiety, and attachment avoidance were associated with reduced resilience. Moreover, the death anxiety-resilience link was not significant for individuals reporting high or low levels of both attachment anxiety and avoidance. The findings are discussed through the prism of Terror Management Theory, and practical implications are suggested.

## 1. Introduction

The aging process is associated with various physical, cognitive, and emotional changes. While most older adults tend to adjust to such declines and continue to enjoy their lives, some find it more difficult to acclimate to age-related difficulties [1]. Moreover, as we age, we become increasingly aware of the fact that our time on earth is not unlimited and that even if we adhere to health recommendations and exercise regularly, we will ultimately die. According to the Terror Management Theory [2], this awareness of our mortality results in detrimental psychological consequences among young and old alike, and research has indeed linked death anxiety in older adults to reduced quality of life, physical health, and increased depression [3,4]. However, little is known about the manner by which such existential concerns affect older adults’ resilience, which has been shown to serve as an important personal resource linked with reduced distress in this age group [5]. Accordingly, the first aim of this study is to examine the associations between death anxiety and resilience among older adults.

As will be elaborated, Terror Management Theory asserts that one of the main defense mechanisms that are employed to ward off death-related thoughts is the ability to seek, maintain, and gain comfort from close relationships [6]. This ability, examined through the tenets of attachment theory and attachment patterns [7], has been linked with a myriad of psychological benefits across childhood and early adulthood, notwithstanding enhanced emotional regulation, life satisfaction, and reduced psychopathology [8]. However, despite the breadth of knowledge pertaining to the importance of attachment patterns for many aspects of human psychological life and its pivotal role in resilience [9], attachment research is relatively lacking in older populations [10]. This issue becomes particularly intriguing when we consider Carstensen’s [11,12] theoretical stance on the growing importance of meaningful close relationships in older age. Moreover, in the current context, it is still unclear whether attachment abilities mitigate the detrimental effects of death anxiety on personal factors that assist older adults in coping with age-related changes and losses. Accordingly, an additional aim of the current work is to examine whether attachment patterns moderate the link between death anxiety and resilience in older adults.

Resilience has been defined by the American Psychological Association [13] as “the process of adapting well in the face of adversity, trauma, tragedy, threats, or significant sources of threat”. In other words, the APA perceives resilience as an internal human capacity that facilitates adjustment and emotional functioning when facing various life events and challenges. Accordingly, research has demonstrated that resilience is an important protective factor against various negative psychological outcomes, such as mood disorders [14], post-traumatic stress disorder [15], and general psychological well-being [16]. More importantly, longitudinal research indicates a causal relationship between existential concerns and resilience. For example, Bennett et al. [17] found that increased death-related thoughts predicted subsequent reduced resilience, and similar findings were reported by Eshel et al. [18] upon examining risk factors for resilience among Israelis exposed to war/terror experiences.

Resilience also seems to play a key role in close social relationships. For example, the quality of children’s friendships was associated with resilience, whereas the quantity of friendships was not [19]. In adult populations, resilience is positively linked with various aspects of perceived social support (i.e., support from friends, family members, and significant others), which was also found to mediate the link between resilience and psychological well-being [20]. Interestingly, there is evidence of a causal relationship between social support and resilience, as receiving support from one’s friends predicted higher resilience 12 months later [21]. This association has also been demonstrated in longitudinal designs, as a sense of belonging and social support predicted increased resilience [17].

It should be noted that the majority of research concerning resilience has tended to focus on specific populations that had been deemed susceptible to the adverse effects of trauma (e.g., at-risk children; military veterans; [22]). However, it seems that the importance of resilience does not diminish with age, and it remains a key factor for successful aging, psychological well-being, and longevity [22,23]. For example, in their meta-analysis, Wermelinger Ávila et al. [24] found an inverse association between resilience and depression among older adults, and resilience was found to moderate the link between childhood trauma and older adults’ depressive symptoms [25]. Moreover, Taylor and Carr [26] found that resilience was linked with reduced functional limitations and the ability to engage in everyday activities, which, in turn, are important factors for social contact with one’s friends and family [27]. In this regard, social support is seen as a major component of resilience, and the ability to recognize, seek, and benefit from close relationships is a key factor for both social support and resilience [28] This stance is also corroborated by research, as high resilience in older populations was linked with social network sizes, frequency of social contact, social support, and the ability to join other people in social gatherings [29,30].

While close relationships are undoubtedly crucial for psychological well-being throughout life, it seems that their importance increases during the aging process. According to the Socioemotional Selectivity Theory (SST) [11,12], younger adults’ relationships tend to focus on knowledge acquisition and establishing social connections which will assist goal achievement (e.g., finding a spouse; career enhancement). As time horizons grow shorter, older adults’ relationship motives undergo a shift from connections aimed at future goal achievement to relationships that aspire to provide meaning in life and increase emotional intimacy [31]. This theoretical stance enunciates the importance of examining not only older adults’ social contacts with others but also their ability to achieve emotional closeness through such contacts. This line of thought is corroborated by research. For example, Lahar et al. [32] report that social support and meaning-making were key factors for older adults dealing with COVID-19-related isolation and social restrictions, stressing the importance of these factors for older adults’ well-being.

Notwithstanding the aforementioned psychological benefits of social connections throughout the life cycle, Agashe et al. [33] claim that social interactions are also important for reducing existential anxiety associated with death (see also [34]). This suggestion can be further understood through the theoretical tenets of Terror Management Theory (TMT; [2]). According to TMT, the unique human capacity to be aware of one’s own existence and the passage of time brings on the realization that death is inevitable. This awareness of our mortality creates a potential for existential terror and intolerable distress, which significantly interferes with our ability to engage in everyday activities. In order to ward off such difficult death-related thoughts and feelings, TMT claims that several defense mechanisms are activated when mortality awareness becomes salient [35]. Such defenses include cultural worldview validation or the sense that individuals are conducting their lives in accordance with cultural values which, in turn, enhances self-esteem and infuses their lives with meaning.

By banking on studies that demonstrate links between death awareness and increased desires for social interactions, romantic intimacy, love, and closeness to one’s life partner [36,37], Mikulincer et al. [7] claimed that the ability to seek, formulate, and maintain close relationships constitutes an additional important death-anxiety buffering mechanism. By utilizing attachment theory as a basis for these abilities [8], Mikulincer and Florian [38] found that individuals with high levels of attachment anxiety (who experience increased fears of abandonment and separation due to the perceived unavailability of the attachment figure) were more affected by death reminders when compared with those with low attachment anxiety. A similar finding was reported for individuals with high levels of attachment avoidance (characterized by a reduced capacity and desire for emotional closeness and high self-reliance). Moreover, for individuals with low anxiety/avoidance (i.e., secure attachment), death awareness resulted in an increased desire to care for others [39], as well as enhanced attachment needs [38]. Additionally, studies examining the links between subjective perceptions of aging and attachment found that secure attachment mitigated the adverse effect of feeling close to one’s death (or subjective nearness-to-death) on negative attitudes toward individuals with disabilities [40], as well as the effect of feeling older than one’s chronological age and loneliness in older adults [41].

In light of the links between death awareness/concerns, attachment abilities, and resilience, the current study sought to examine whether attachment patterns moderate the association between death anxiety and resilience in older adults. Three hypotheses were formulated: (1) high levels of death anxiety will be associated with reduced resilience; (2) high levels of attachment anxiety and avoidance will be associated with reduced resilience; and (3) attachment patterns would moderate the death anxiety-resilience link, as this association would not be significant in older adults displaying low levels of attachment anxiety/avoidance.

## 2. Materials and Methods

### 2.1. Participants and Procedure

Data were collected between December, 2022, and March, 2023, from a convenience sample of 369 Israeli older adults (age range 60–94, *M* = 73.15, *SD* = 6.31), of which 130 (35.2%) were men. Most participants were community-dwelling (*n* = 289; 78.3%) and were in a relationship (*n* = 263; 71.3%; see Table 1 for means, SDs, and correlation matrix for the study variables). The inclusion criteria included being over the age of 60 adequate knowledge of Hebrew, and computer literacy.

Research assistants approached eligible participants through snowball sampling. After verifying that they met the inclusion criteria (e.g., assessing language skills and computer literacy through email exchanges), participants were informed about the study aims and provided informed consent. Subsequently, participants were provided with a designated Qualtrics link (through email/WhatsApp), which led to the study scales (see below). Participants were also asked to provide sociodemographic and background information such as age, gender (male/female), relationship status (not in a relationship/in a relationship), and self-rated economic status (examined by an item ranging from 1, “not very good”, to 5, “very good”). Moreover, participants reported whether they lived in the community and were asked to indicate whether they had been diagnosed with one or more of 15 medical conditions (e.g., cardiovascular illnesses; cancer; see [42]). As these variables were found to be potential covariates for the study variables in previous studies [43,44,45], they were controlled in the analyses. The study was approved by the institutional review board of Ashkelon Academic College.

### 2.2. Measures

Death anxiety was examined by six relevant items (e.g., “I am very afraid of death”) provided in Carmel and Mutran’s [46] questionnaire. These items are rated on a scale ranging from 1 (“completely disagree”) to 5 (“completely agree”). A mean score was calculated for each participant, and higher scores indicate high levels of death anxiety. The scale has demonstrated high reliability [43], and Cronbach’s alpha in the current study was 0.91.

Resilience was assessed by the 10-item validated version of the Connor-Davidson Resilience Scale (CD-RISC; [47,48]). This self-report scale measures resilience (e.g., “I am not easily discouraged by failure”) on a Likert scale ranging from 0 (“not true at all”) to 4 (“extremely true”). The total score was calculated by summing all 10 items, and higher scores indicate higher resilience. Cronbach’s alpha in the current study was 0.87.

Attachment patterns were assessed by the Experiences in Close Relationships scale [49]. This scale comprises 36 items, 18 of which examine attachment anxiety (e.g., “I worry about being abandoned”) and 18 focus on attachment avoidance (e.g., “I prefer not to show people how I feel deep down”). Each item is rated on a scale ranging from 1, “strongly disagree”, to 7, “strongly agree”. Two mean scores are calculated, one for attachment anxiety (α = 0.90) and one for attachment avoidance (α = 0.85).

### 2.3. Data Analysis

Analyses were conducted using the SPSS 24 software. Prior to the examination of the hypotheses, initial correlations between the study variables were calculated (see Table 1 for means, SDs, and correlation matrix for the cohort). The study hypotheses were examined by a hierarchical regression, with resilience as the outcome variable. The first step included the covariates of age, gender (male/female), relationship status (not in a relationship/in a relationship), self-rated economic status, living arrangement (community-dwelling/other), and the sum of medical conditions. The second step included the main effects of death anxiety, attachment anxiety, and attachment avoidance. The third step included the three possible two-way interactions (death anxiety × attachment anxiety, death anxiety × attachment avoidance, and attachment anxiety × attachment avoidance), in order to control for the potential confounding effects of these interactions on the hypothesized three-way interaction. The fourth and final step included the three-way interaction of death anxiety × attachment anxiety × attachment avoidance. Significant interactions were probed using Model 3 of the PROCESS 4.2 macro for SPSS [50].

In order to further confirm the study model’s reliability and validity, two additional analyses were conducted prior to the examination of the study hypotheses. First, we performed confirmatory factor analyses for the four study variables (death anxiety, attachment anxiety, attachment avoidance, and resilience) and found a good model fit for all constructs. Regarding validity, we employed the Fornell–Larcker criterion [51] and found that in line with the requirements, all four Average Variance Extracted (AVE) values were higher than the correlations between the study variables. Moreover, a power analysis for detecting a medium effect size (0.15) with 13 predictors required a sample size of 189, indicating sufficient power for the current sample. Additionally, potential multicollinearity was rejected, as tolerance and VIF ranges (0.64–0.96; 1.04–1.57, respectively) for the regression analysis were in line with the literature requirements [52].

## 3. Results

The initial correlations between the study variables demonstrated that high death anxiety was associated with higher levels of attachment anxiety (r = 0.53, *p* < 0.001), attachment avoidance (r = 0.12, *p* < 0.05), and lower levels of resilience (r = −0.32, *p* < 0.001; see Table 1). Attachment anxiety was associated with higher attachment avoidance (r = 0.28, *p* < 0.001) and reduced resilience (r = −0.46, *p* < 0.001), and attachment avoidance was also linked with reduced resilience (r = −0.40, *p* < 0.001).

**Figure 1:**
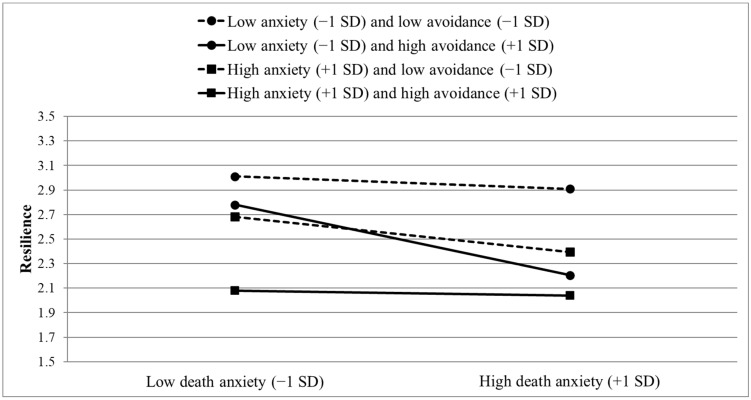
The three-way interaction between death anxiety, attachment anxiety, and attachment avoidance in predicting resilience.

In line with the first hypothesis, the regression analysis for resilience revealed a main effect for death anxiety (*B* = −0.81, *SE* = 0.39, β = −0.11, *p* < 0.05; see Table 2), indicating that high death anxiety levels were associated with reduced resilience. Moreover, in line with the second hypothesis, similar main effects were found for attachment anxiety (*B* = −2.03, *SE* = 0.36, β = −0.30, *p* < 0.001) and attachment avoidance (*B* = −2.28, *SE* = 0.37, β = −0.28, *p* < 0.001), indicating that high levels of attachment anxiety/avoidance were associated with reduced resilience. Finally, in accordance with the third hypothesis, the three-way interaction of death anxiety × attachment anxiety × attachment avoidance was significant (*B* = 0.85, *SE* = 0.30, β = 0.17, *p* < 0.01), adding 1.5% to the explained variance of the model (total R^2^ = 0.377). Probing the interaction using PROCESS [50] revealed that the connection between death anxiety and resilience remained significant for individuals with low anxiety/high avoidance (*B* = −2.88, *SE* = 0.91, β = −0.37, *p* < 0.01) and high anxiety/low avoidance (*B* = −1.45, *SE* = 0.60, β = −0.20, *p* < 0.001). However, this effect was nullified for subjects reporting low levels of anxiety/avoidance (*B* = −0.50, *SE* = 0.67, β = −0.06, *p*> 0.05) and for those reporting high levels of anxiety/avoidance (*B* = −0.02, *SE* = 0.57, β = −0.01, *p* > 0.05; see Figure 1). It should be noted that the results remained unchanged when the covariates were excluded from the analysis.

## 4. Discussion

Resilience is considered to be a vital personal resource that increases individuals’ capability of coping with various stressors and adversity throughout the life cycle, thereby enhancing psychological well-being [14,15,16]. Moreover, resilience also promotes positive social relationships and is seen as a protective factor against existential anxieties concerning death and dying [33]. However, research in this field has been somewhat lacking among older populations, in which awareness of one’s mortality may become more salient [53]. In light of theoretical stances that delineate the increased importance of social relationships in older adulthood [11,12], as well as the role of the ability to form and gain comfort from such relationships in mitigating existential concerns [36,37,38], we sought to examine the links between death anxiety, attachment patterns, and resilience in older adults.

In line with the first hypothesis, high levels of death anxiety were associated with reduced resilience. This finding is in line with studies confirming the inverse connection between existential concerns and protective psychological factors in older adults, such as emotional complexity [54], self-esteem, and a sense of meaning in life [55]. Moreover, it corresponds with Rayatpisheh et al. [56], who found that during the COVID-19 pandemic, older adults’ death anxiety was indicative of reduced resilience. Interestingly, while Cicirelli [57] found that death anxiety was more pronounced in younger adults when compared to older adults, our findings demonstrate that regardless of this potential age-related decline, death concerns are nevertheless linked with reduced personal resources in the latter group.

The second hypothesis was also confirmed, as high levels of both attachment anxiety and attachment avoidance were associated with reduced resilience. According to Darling Rasmussen et al. [9], attachment behavior is closely linked with the ability to manage adverse life events and stressors, and secure attachment (i.e., low anxiety/avoidance) “might be the core factor or prerequisite in positive adaptation” (p. 1288), which is a cornerstone of resilience (see also [58]). Moreover, Magai et al. [10] argue that older adults with high levels of attachment anxiety/avoidance may find it more difficult to deal with their declining autonomy and to seek emotional and instrumental assistance from their children. Accordingly, our findings are in line with the theoretical stance of SST [11,12], which, as previously noted, claims that when time horizons are perceived to be long and open-ended, social networks are targeted toward gaining knowledge and obtaining goals. However, when time horizons are seen as limited, older adults maintain emotional well-being by investing in relationships that provide closeness and meaning to their lives. As attachment research in older adults is relatively lacking [10], our findings provide an important understanding of how older adults’ abilities to seek, maintain, and benefit from close relationships are associated with resilience.

Apart from the beneficial psychological consequences of secure attachment (see review by [8]), secure attachment has also been found to mitigate the adverse psychological consequences of death awareness and concerns in younger adults [6,38]. Accordingly, it was hypothesized that attachment patterns would moderate the link between death anxiety and resilience in older adults. This hypothesis was confirmed, as the aforementioned link was not significant among individuals reporting low levels of both anxiety and avoidance (i.e., secure attachment). While attachment behaviors in older adults have been examined vis-à-vis older adults’ relationships with their children and their ability to ask for and receive assistance from them [10], little was known about how attachment patterns may mitigate the positive association between death anxiety and personal resources. In accordance with TMT [2] and with the understanding of the important role of social relationships in mitigating the adverse effects of death-related thoughts and concerns in younger cohorts, it can be stated that this utility does not diminish with age.

However, if we follow this line of thought, the nullification of the death anxiety-resilience link in older adults reporting *high* levels of both anxiety and avoidance seems incompatible with the manner by which TMT conceives the utility of attachment patterns for mitigating existential concerns. One possible explanation may stem from the fact that high levels of both anxiety and avoidance were linked with increased neuroticism and reduced conscientiousness in adults [59], as well as with reduced ego-resiliency, or the ability to regulate one’s cognitive/emotional behaviors in response to situational challenges [60]. Moreover, Mikulincer [6] claims that anxiously attached individuals display higher levels of fear of death and heightened accessibility of death-related thoughts even with the absence of a specific trigger, whereas those with high avoidance tend to report high death anxiety on projective measures, but do not display heightened death anxiety when examined explicitly. Thus, it may be suggested that the discrepancy between disorganized individuals’ strong desire for close relationships (as portrayed by high anxiety), together with their deep distrust of others and need for self-reliance (as indicated by high avoidance), may contribute to low levels of resilience, which remain unaffected by existential concerns. While the results seem to provide initial evidence for this line of reasoning (as can be seen in the low levels of resilience among individuals with high anxiety/avoidance in Figure 1, regardless of death anxiety levels), additional evidence is required to elucidate this issue.

Several limitations of the current work should be noted. First and foremost, the cross-sectional nature of this study precludes the possibility of inferring causal connections between the study variables. Although the study model is backed by the theoretical assumptions of TMT, it is important to examine the study model longitudinally. Second, the cohort comprised relatively healthy and educated Israeli older adults, and this, for example, may hinder the generalizability of the results to older adults displaying reduced health and/or physical difficulties, which may lead to increased social isolation and difficulties in maintaining close relationships. In this regard, the online nature of the study hindered the possibility of verifying the information provided by the participants, and this issue should be considered in future examinations of the study model. Moreover, while the study was conducted during a relatively peaceful time in Israel, we do not have information about the participants’ area of residence. Consequently, as some participants may live in close proximity to border areas in Israel, in which the threat of terror attacks may be a daily concern, this may affect death anxiety and resilience levels.

Despite these limitations, the study offers a unique insight into how older adults utilize their attachment abilities for buffering the adverse effect of death anxiety, and into how this ability affects their resilience. The findings may be of relevance to healthcare practitioners who strive to enhance older adults’ personal resources in order to assist them in dealing with difficulties associated with the aging process. For example, clinical programs/interventions that aim to enhance older adults’ resilience are encouraged to consider death anxiety as a contributing factor in this context. Moreover, clinicians who encounter existential concerns and fears are encouraged to explore not only the quantity of older clients’ close relationships but also their ability to benefit from these relationships, by implementing the tenets of attachment theory into their clinical work. By demonstrating the underlying mechanisms of older adults’ positive social relationships through attachment theory, attachment-based interventions may be beneficial not only for increasing older adults’ quality of life but also for mitigating the harmful psychological effects of existential concerns and fears.

## Figures and Tables

**Table 1:** Means, SDs, and correlation matrix for the cohort.

	Variable	M/%	SD	1	2	3	4	5	6	7	8	9
1.	Age	73.15	6.31	--								
2.	Gender ^a^ (male)	35.2%		0.02	--							
3.	Relationship status ^b^ (not in a relationship)	28.7%		−0.28 ***	−0.13 *	--						
4.	Economic status	3.62	0.92	−0.02	0.08	0.22 ***	--					
5.	Living arrangement ^c^ (community)	78.3%		0.01	−0.05	−0.15 **	−0.10	--				
6.	Medical conditions	2.16	1.15	0.18 ***	0.06	−0.05	−0.12 *	−0.02	--			
7.	Death anxiety	2.26	0.93	0.03	0.04	−0.06	−0.19 ***	0.02	0.04	--		
8.	Attachment anxiety	3.27	1.03	0.09	0.01	−0.14 **	−0.19 ***	−0.09	0.15 **	0.53 ***	--	
9.	Attachment avoidance	3.43	0.86	−0.02	−0.09	−0.13 *	−0.17 **	0.02	0.08	0.12 *	0.28 ***	--
10.	Resilience	25.32	7.05	−0.13 *	−0.04	0.28 ***	−0.17 **	−0.09	−0.17 **	−0.32 ***	−0.46 ***	−0.40 ***

Notes: *n* = 369. Correlation values represent Pearson coefficients except for coefficients for gender, relationship status, and living arrangement, which represent point-biserial coefficients. ^a^—0 = male; 1 = female. ^b^—0 = not in a relationship; 1 = in a relationship. ^c^—0 = community-dwelling; 1 = else (e.g., assisted living facilities). * = *p* < 0.05; ** = *p* < 0.01; *** = *p* < 0.001.

**Table 2:** Hierarchical regression coefficients for resilience (*n* = 369).

			95% CI for *B*						
	Variable	*B*	*LL*	*UL*	*SE B*	β	t	*p*	ΔR^2^
Step 1									0.111 ***
	Age	−0.03	−0.15	0.09	0.06	−0.03	−0.51	0.609	
	Gender ^a^	−0.21	−1.70	1.27	0.75	−0.01	−0.28	0.781	
	Relationship status ^b^	3.65	1.94	5.37	0.87	0.23	4.19	<0.001	
	Economic status	0.73	−0.06	1.52	0.40	0.10	1.82	0.069	
	Living arrangement ^c^	−0.66	−2.39	1.06	0.87	−0.04	−0.76	0.448	
	Medical conditions	−0.84	−1.46	−0.22	0.32	−0.14	−2.66	0.008	
Step 2									0.246 ***
	Death anxiety	−0.81	−1.57	−0.05	0.39	−0.11	−2.11	0.036	
	Attachment anxiety	−2.03	−2.75	−1.31	0.36	−0.30	−5.55	<0.001	
	Attachment avoidance	−2.28	−3.02	−1.54	0.37	−0.28	−6.09	<0.001	
Step 3									0.005
	Death anxiety × Attachment anxiety	0.42	−0.15	0.99	0.29	0.07	1.44	0.150	
	Death anxiety × Attachment avoidance	0.07	−0.57	0.71	0.33	0.01	0.21	0.833	
	Attachment anxiety × Attachment avoidance	0.05	−0.58	0.69	0.32	0.01	0.17	0.865	
Step 4									0.015 **
	Death anxiety × Attachment anxiety × Attachment avoidance	0.85	0.26	1.45	0.30	0.17	2.84	0.005	
Total R^2^									0.377

Notes: ^a^—0 = male; 1 = female. ^b^—0 = not in a relationship; 1 = in a relationship. ^c^—0 = community-dwelling; 1 = else (e.g., assisted living facilities). CI = confidence interval; *LL* = lower limit; *UL* = upper limit; ** = *p* < 0.01; *** = *p* < 0.001.

## Data Availability

Data are available from the author upon reasonable request.
